# Inhibition of PP2A with LB-100 Enhances Efficacy of CAR-T Cell Therapy Against Glioblastoma

**DOI:** 10.3390/cancers12010139

**Published:** 2020-01-06

**Authors:** Jing Cui, Herui Wang, Rogelio Medina, Qi Zhang, Chen Xu, Iris H. Indig, Jingcheng Zhou, Qi Song, Pauline Dmitriev, Mitchell Y. Sun, Liemei Guo, Yang Wang, Jared S. Rosenblum, John S. Kovach, Mark R. Gilbert, Zhengping Zhuang

**Affiliations:** 1 Neuro-Oncology Branch, Center for Cancer Research, National Cancer Institute, National Institutes of Health, Bethesda, MD 20892, USA; 2 Surgical Neurology Branch, National Institute of Neurological Disorders and Stroke, National Institutes of Health, Bethesda, MD 20892, USA; 3 David Geffen School of Medicine, University of California, Los Angeles, CA 90095, USA; 4 Lixte Biotechnology Holdings, Inc., East Setauket, NY 11733, USA

**Keywords:** CAR-T, LB-100, CAIX, PP2A, glioblastoma

## Abstract

Chimeric antigen receptor (CAR)-engineered T cells represent a promising modality for treating glioblastoma. Recently, we demonstrated that CAR-T cells targeting carbonic anhydrase IX (CAIX), a protein involved in HIF-1a hypoxic signaling, is a promising CAR-T cell target in an intracranial murine glioblastoma model. Anti-CAIX CAR-T cell therapy is limited by its suboptimal activation within the tumor microenvironment. LB-100, a small molecular inhibitor of protein phosphatase 2A (PP2A), has been shown to enhance T cell anti-tumor activity through activation of the mTOR signaling pathway. Herein, we investigated if a treatment strategy consisting of a combination of LB-100 and anti-CAIX CAR-T cell therapy produced a synergistic anti-tumor effect. Our studies demonstrate that LB-100 enhanced anti-CAIX CAR-T cell treatment efficacy in vitro and in vivo. Our findings demonstrate the role of LB-100 in augmenting the cytotoxic activity of anti-CAIX CAR-T cells and underscore the synergistic therapeutic potential of applying combination LB-100 and CAR-T Cell therapy to other solid tumors.

## 1. Introduction

Glioblastoma is the most common malignant primary brain tumor in humans and is associated with a dismal prognosis [1,2]. The present standard of care, consisting of maximal safe tumor resection, radiotherapy, and temozolomide, has modestly increased median and short-term survival rates [3]. Despite these improvements, the 5-year survival of patients remains at approximately 10% [4]. Considering the status quo, it is clear that more effective treatments for glioblastoma are urgently needed.

Recently, chimeric antigen receptor (CAR) T therapy has demonstrated strong therapeutic potential in the treatment of glioblastoma. Several CAR-T treatments utilizing NKG2D, CD70, EGFRvIII, IL13Rα2, HER2, and EphA2 as targets have been studied for glioblastoma [5,6,7,8,9,10,11,12]. In particular, the EGFRvIII, IL13Rα2, HER2-targeted CAR-T therapy demonstrated promising results in the clinical setting [5,10,11,12,13]. In addition, we previously showed that CAR-T cells targeting Carbonic anhydrase IX (CAIX), a membrane-bound protein involved in maintaining intracellular pH that is upregulated in glioblastoma tumor cells, curtailed tumor growth and improved survival in glioblastoma-bearing mice [14,15].

However, several challenges still stand in optimizing CAR-T therapy’s potential in glioblastoma and other solid tumors, including the limited penetration of CAR-T cells into solid tumors and their sub-optimal function within immunosuppressive microenvironments [16]. Recently, several combinations of immunotherapies and CAR-T cell therapy have been attempted to achieve improved tumor control [17]. Here, we investigated if the efficacy of the anti-CAIX CAR-T cells could be augmented when combined with an immune-modulating agent.

Protein phosphatase-2A (PP2A) is a ubiquitous serine/threonine phosphatase that negatively regulates cytotoxic T-cell effector function [18]. Previously, we demonstrated that pharmacologic inhibition of PP2A by LB-100 could enhance immune activation by activating mTORC1 pathways [19]. A recently completed Phase I clinical trial demonstrated LB-100, the first-in-class small molecular inhibitor of PP2A, was well tolerated by adult patients with progressive solid tumors [20]. Considering the established pharmacological tolerability and efficacy of LB-100 in augmenting the effector activities of T-lymphocytes and its established pharmacological tolerability, further investigation into its ability to enhance CAR-T cell immunotherapy against solid tumors is warranted. 

In the present study, we studied the ability of LB-100 to enhance CAIX targeting CAR-T cell effectiveness in a glioblastoma xenograft mouse model. We showed that the efficacy of CAR-T cell therapy against glioblastoma is augmented when combined with LB-100, underscoring its translational potential to other solid tumors.

## 2. Results

### 2.1. LB-100 Enhances Cytotoxicity of Anti-CAIX CAR-T Cells In Vitro

Previously, we reported that inhibition of PP2A by LB-100 enhanced the cytotoxic T cell activity in a murine colon cancer model [19]. To investigate if LB-100 similarly enhanced the cytotoxicity profile of the anti-CAIX CAR-T cell, we performed a series of in vitro experiments using four glioblastoma cell lines (T98G, A172, LN229, and U251). As we previously reported, U251 demonstrated more sensitivity to hypoxia-induced CAIX expression when compared to the other three cell lines [14]. Here, we also evaluated the U251-Luc glioblastoma cell line, which can be used to generate an orthotopic xenograft mouse model for in vivo study. 

We first investigated the impact of LB-100 on CAR-T cells alone. Anti-CAIX CAR-T cells were generated as previously reported [14]. The transduction efficiency was evaluated using a mock vector (containing a green fluorescent protein-GFP expression element instead of CAIX scFv sequences) transduced T cells and assessed by GFP expression 4 days post-transduction. Flow cytometry analysis showed that approximately 30% of T cells expressed GFP (Figure S1A). Empty vector transduced T cells were utilized as control T cells in the following study. We tested the effect of LB-100 on the viability of T cells after 48 h of drug treatment using CCK-8 assays. The 50% inhibitory concentration (IC50) of LB-100 was 8.42, 8.34, and 8.19 µM for non-transduced T, control T, and anti-CAIX CAR-T cells, respectively (Figure S1B). Treatment with LB-100 at a dose above 1 µM reduced the viability of T cells. In contrast, T cells treated with LB-100 at a dose below 1 µM showed no significant increase in proliferation. Next, we assessed the effect of LB-100 on the cell surface expression of CAR by protein L (CAR scFv marker) as determined by flow cytometry. The anti-CAIX CAR expression was comparable in CAR-T cells treated with 1 µM of LB-100 for 48 h and un-treated CAR-T cells (Figure S1C,D).

Next, we evaluated the cytotoxicity of LB-100 in glioblastoma cell lines alone. A dose-response curve using CCK-8 assays after 48 h of drug treatment demonstrated dose-dependent inhibition of cell growth in all the cell lines. The IC50 of LB-100 was 6.85, 6.39, 6.19, 10.88, and 10.01 µM for T98G, A172, LN229, U251, and U251-Luc cells, respectively (Figure S2A). The viabilities of glioblastoma cells treated with LB-100 at a dose below 1 µM were not significantly affected. Considering these findings, we used graded doses of LB-100 below 1 µM in subsequent studies to assess LB-100’s immune-modulating effect independent of an intrinsic cancer response to LB-100 and to avoid its cytotoxicity on T cells. 

**Figure 1:**
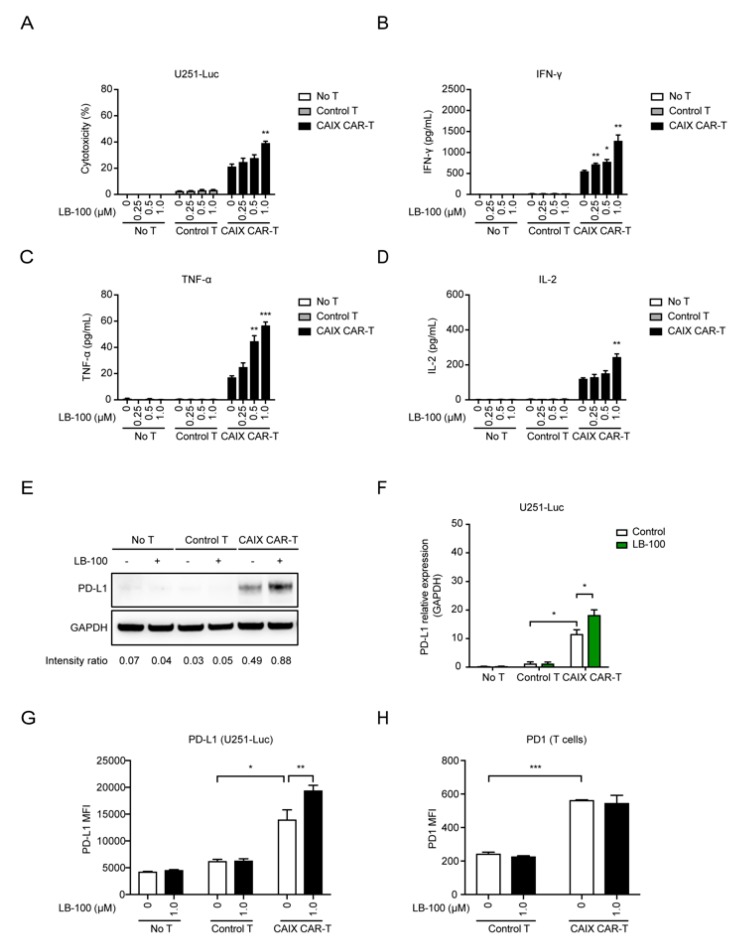
LB-100 enhances cytotoxicity of anti-carbonic anhydrase IX chimeric antigen receptor-T (anti-CAIX CAR-T) cells in vitro. (**A**) Control T cells (empty vector transduced T cells) or anti-CAIX CAR-T cells were co-cultured with U251-Luc cells with titration concentration of LB-100 as indicated (1 µM) at an E/T ratio at 4 for 48 h. Cytotoxicity was determined by LDH releasing assay. *n* = 3 for each group. (**B**–**D**) Levels of IFN-γ (**B**), TNF-α (**C**), and IL-2 (**D**) secretion in the supernatant obtained from the cocultured system were analyzed by ELISA. The bar graphs represent a significant increase in cytokine release in anti-CAIX CAR-T treated groups. A combination of LB-100 further enhanced cytokine release. (**E**,**F**) Representative Western blots showed increased expression of PD-L1 in anti-CAIX CAR-T treated groups, especially in the combination groups, compared to control T cell treated groups and untreated groups (*n* = 3 for each group). (**F**) A quantitative comparison is listed. The expression of GAPDH served as the internal control to calculate relative expression levels. (**G**) Flow cytometry analyzing PD-L1 expression on untreated, control T cell treated, and anti-CAIX CAR-T cell treated U251-Luc cells in the presence of 1 µM LB-100 (*n* = 3). There is a significant increase in mean fluorescence intensity (MFI) of PD-L1 positive cells in anti-CAIX CAR-T treated groups, especially in the combination groups. (**H**) Flow cytometry analyzing PD-1 expression on control T cells and anti-CAIX CAR-T cells co-cultured with U251-Luc cells in the presence of 1 µM LB-100 (*n* = 3). There is a significant increase in mean fluorescence intensity (MFI) of PD-1 positive cells in anti-CAIX CAR-T cells compared with control T cells. LB-100 has little effect on PD-1 expression of T cells. All data are shown as the mean ± SEM. * *p* < 0.05, ** *p* < 0.01, and *** *p* < 0.001 by Student’s *t*-test, anti-CAIX CAR-T combined with LB-100 groups vs. anti-CAIX CAR-T group; anti-CAIX CAR-T groups vs. control T groups in (F–H).

To assess in vitro functionality of the anti-CAIX CAR-T cells combined with LB-100 against glioblastoma cells, tumor cells were co-cultured with anti-CAIX CAR-T cells or control T cells and treated with titration of LB-100 for 48 h. As we previously demonstrated, anti-CAIX CAR-T cells showed dose-dependent cytotoxicity on U251 cells in different effector (E):target (T) ratios, ranging from 1:5 to 5:1 [14]. Higher E:T ratio showed more significant cytotoxicity. Thus, we performed combination treatment against glioblastoma cells with CAR-T cells at a relative high E:T ratio of 4:1. We evaluated the cytotoxicity and cytokine production (IFN-γ, TNF-α, and IL-2) of anti-CAIX CAR-T cells combined with LB-100 in the co-culture system. We detected significant cytotoxicity against glioblastoma cells and increased levels of IFN-γ, TNF-α, and IL-2 in the presence of anti-CAIX CAR-T cells but not in control T cells (Figure 1A–D and Figure S3A–B). LB-100 significantly increased the cytotoxic effect of the anti-CAIX CAR-T cell (Figure 1A and Figure S3A) in a dose-dependent manner. Consistently, we noted a significant increase in the production of IFN-γ, TNF-α, and IL-2 by the anti-CAIX CAR-T cells in the presence of LB-100 (Figure 1B–D and Figure S3B). However, LB-100 has little effect on cytokine production in control T treated glioblastoma cells, glioblastoma cultured alone, and T cells cultured alone, respectively (Figure 1B–D and Figure S3B,C). These results indicated that LB-100’s synergistic effect is associated with antigen-specific cytotoxicity and suggests that LB-100 plays a significant role in enhancing the anti-tumor activity of anti-CAIX CAR-T cells.

Previous studies reported that the interaction of checkpoint molecule-programmed death-1 (PD-1) and its ligand-PD-L1 may partly contribute to the failure of CAR-T cell therapy [21,22,23]. The enhanced production of IFN-γ in the tumor microenvironment induced an increase in PD-L1 expression in the tumor that attenuates tumor immunity in other studies [24,25,26]. Thus, evaluating the PD-L1 expression in tumor cells after exposure to CAR-T cells may help measure the efficacy of CAR-T cell therapy in preclinical study. Considering that LB-100 significantly elevated the cytotoxicity profile of the anti-CAIX CAR-T cells, we investigated if combining anti-CAIX CAR-T cells with LB-100 induced increased expression of PD-L1 in U251-Luc cells when compared to either treatment alone. U251-Luc tumor cells were co-cultured with anti-CAIX CAR-T cells or control T cells and treated with 1 µM of LB-100 for 48 h. Western blot analyses demonstrated that U251-Luc cells treated with a combination of anti-CAIX CAR-T cells and LB-100 had higher expression of PD-L1 compared to U251-Luc cells treated with anti-CAIX CAR-T cells or LB-100 alone (Figure 1E,F and Figure S4). No significant elevation of PD-L1 expression was observed in U251-Luc cells treated with control T cells. Further, flow cytometry analysis of PD-L1 expression on U251-Luc cells was consistent with Western blot results and demonstrated that the combination treatment significantly augmented PD-L1 expression on tumor cells (Figure 1G). We demonstrated that PD-L1 expression on U251-Luc cells treated with anti-CAIX CAR-T cells was stable 24 h after T cell washout (Figure S5A). Furthermore, assessment of PD-L1 expression on T cells revealed a much lower expression when compared to tumor cells. Anti-CAIX CAR-T cells demonstrated a significant increase in both PD-L1 and PD-1 expressions when compared to control T Cells (Figure S5B and Figure 1H). Notably, both anti-CAIX CAR-T cells and control T cells did not demonstrate an increase in PD-L1 or PD-1 expressions when combined with LB-100. 

Collectively, in vitro experimental data suggests that inhibition of PP2A by LB-100 enhances the cytotoxicity of CAR-T cells and augments adaptive immune resistance in CAR-T treated tumor cells via increased expression of PD-L1.

### 2.2. LB-100 Enhances mTORC1 Activation in Anti-CAIX CAR-T Cells

**Figure 2:**
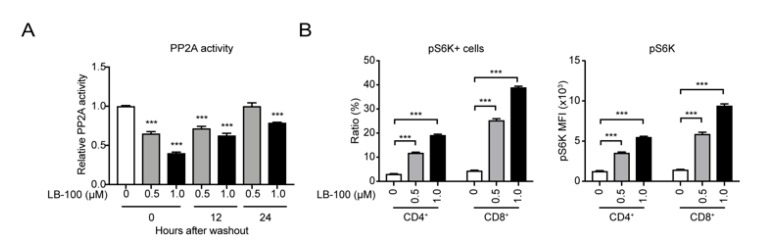
LB-100 suppresses protein phosphatase 2A (PP2A) and activates mTOR signaling in anti-CAIX CAR-T cells. (**A**) Anti-CAIX CAR-T cells were treated with 0.5 µM, 1 µM LB-100 for 6 h, followed by washout for 12 h and 24 h. PP2A enzymatic activity was measured after 6 h in the presence of LB-100 and at the time points after LB-100 washout as indicated. PP2A activity was normalized relative to the activated control without LB-100 treatment (*n* = 3). (**B**) Flow cytometry analyzing phosphorylated S6K (p-S6K) in the presence of LB-100 (*n* = 5). There is a significant increase in ratio and mean fluorescence intensity (MFI) of pS6K positive cells in LB-100 treated CAR-T cells. All data are shown as the mean ± SEM. *** *p* < 0.001 by Student’s *t*-test, anti-CAIX CAR-T combined with LB-100 groups vs. anti-CAIX CAR-T group.

Previously, we demonstrated that inhibition of PP2A with LB-100 enhanced T-cell activity by promoting the mTORC1 signaling pathway [19]. To confirm that LB-100 enhanced the activation of anti-CAIX CAR-T cells through the same mTORC1 mechanism, we assessed the dose-dependent inhibitory effect of LB-100 on PP2A enzyme activity. Anti-CAIX CAR-T cells were treated with LB-100 for 6 h, followed by washout. PP2A assay results showed that there was a significant decrease in PP2A enzymatic activity in CAIX CAR-T cells treated with LB-100 for 6 h, and recovery of PP2A activity was observed in 24 h after washout (Figure 2A). Additionally, flow cytometry analysis demonstrated a dose-dependent increase in activity of mTORC1 as measured by phosphorylation of Ribosomal protein S6 kinase (S6K), which has been used as a hallmark of mTOR activation (Figure 2B). Together, these data suggest that PP2A inhibition enhances the activity of CAIX CAR-T cells by activating the mTORC1 axis.

### 2.3. LB-100 Enhances Anti-CAIX CAR-T Efficacy Against Glioblastoma In Vivo

**Figure 3:**
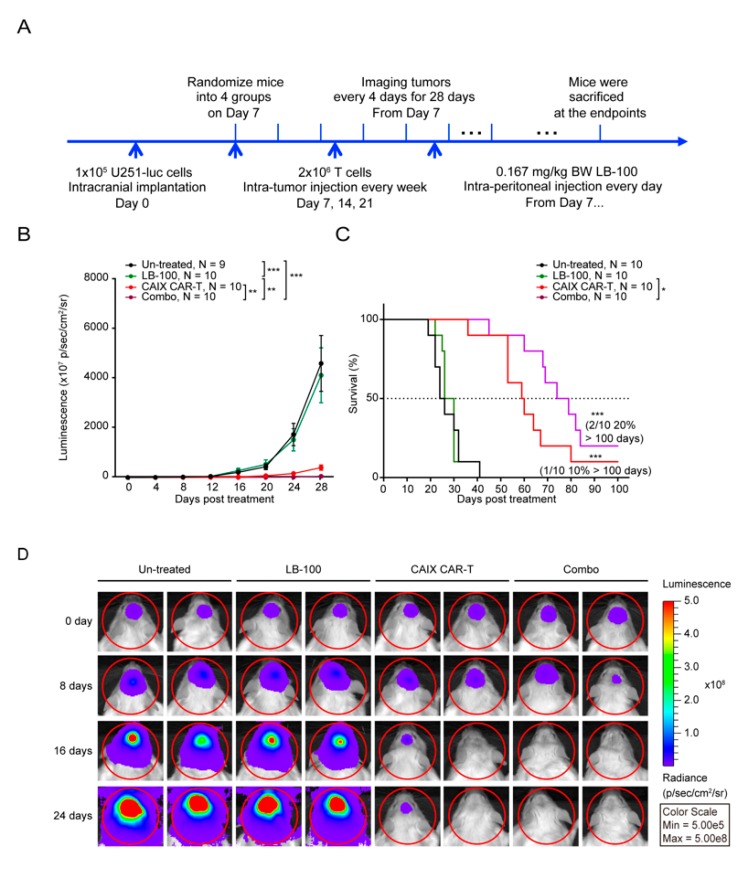
LB-100 enhances anti-CAIX CAR-T efficacy against glioblastoma in vivo. (**A**) The schematic diagram of the progression of the experiment in vivo. One week after 1 × 10^5^ U251-Luc cells were inoculated into the brain of NSG mice, mice were randomized into four groups (*n* = 9–10 for each group): un-treated, LB-100, anti-CAIX CAR-T, and Combo (LB-100 plus anti-CAIX CAR-T). Mice in anti-CAIX CAR-T and Combo treated groups were injected in situ with 2 × 10^6^ anti-CAIX CAR-T cells. LB-100 was administrated into mice in LB-100 and Combo groups daily at a dose of 0.167 mg/kg. Mice were monitored every four days for 28 days via luminescence imaging to follow tumor progression. (**B**) Bioluminescence imaging results showed that the combination of LB-100 resulted in striking regression of tumors compared to LB-100 or anti-CAIX CAR-T alone group. *p*-value was calculated by two-way ANOVA. * *p* < 0.05, ** p < 0.01, *** p < 0.001. (**C**) The survival curve showed that the combination of LB-100 had a significantly prolonged survival compared with either treatment alone. *p*-value was calculated by log–rank test analysis. *** *p* < 0.001. The median survival of the Combo treated group was 76.5 days, compared to 59.5 days, 28 days, and 25 days in the anti-CAIX CAR-T, LB-100, and un-treated control groups, respectively. (**D**) Representative tumor-derived bioluminescence images of U251-Luc tumor bearing mice at indicated time points after T-cell treatment.

To test the hypothesis that PP2A inhibition synergizes with anti-CAIX CAR-T therapy in vivo, we established an intracranial U251-Luc glioma mouse model (Figure 3A). One week after intracranial inoculation, tumor growth was confirmed by in vivo bioluminescence imaging, and then mice were randomized into four treatment groups: Untreated, LB-100, anti-CAIX CAR-T, and Combo (LB-100 plus anti-CAIX CAR-T) groups. In the anti-CAIX CAR-T and Combo groups, mice were injected intracranially with 2 × 10^6^ anti-CAIX CAR-T cells every week for a total of three dosages. In LB-100 and Combo groups, mice were intraperitoneally injected with LB-100, daily, at a low dose of 0.167 mg/kg body weight as previously reported for the target effect on the T cells [19]. Tumor growth was assessed every four days with bioluminescence imaging. 

Bioluminescence imaging results showed that the combination of anti-CAIX CAR-T cells and LB-100 resulted in a striking regression of tumors and a significant increase in survival when compared to control or single treatment groups (Figure 3B,C). Complete regression of tumor was achieved in 20% of combination-treated mice, while 10% of anti-CAIX CAR-T cells alone treated mice, whereas no anti-tumor effects were observed in LB-100 alone treated mice (Figure 3B–D).

To further confirm that LB-100 could enhance CAR-T cell activity, we performed a tumor-infiltrating lymphocyte analysis. Mice were similarly implanted with U251-Luc tumors and randomized into the following four treatment groups: Un-treated, LB-100, anti-CAIX CAR-T, and Combo (LB-100 plus anti-CAIX CAR-T). After two weeks of treatment, brain tumors were harvested and analyzed by flow cytometry with human T cell markers (CD3^+^, CD4^+^, and CD8^+^) according to the previously described gating strategy [14]. 

**Figure 4:**
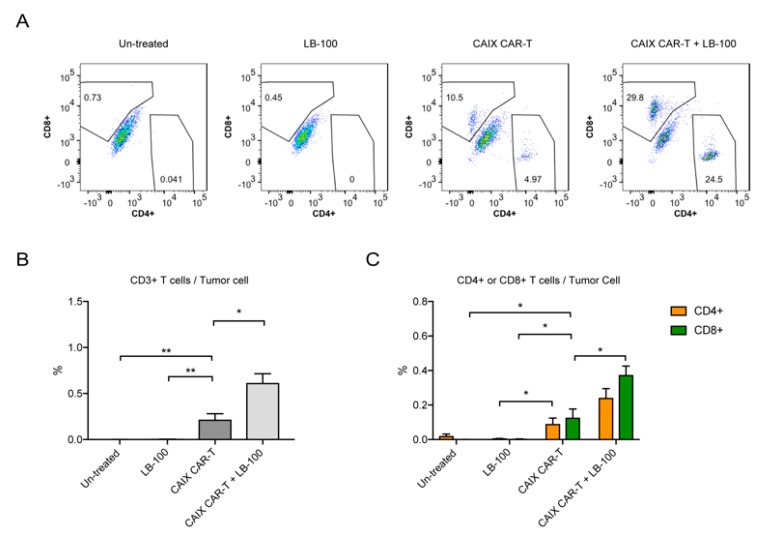
LB-100 increases CAR-T cell quantities in tumors. (**A**–**C**) Tumor-infiltrating lymphocytes (TILs) analysis for the glioblastoma xenograft mouse model was established as described above. Mice were randomized into four groups: Un-treated (*n* = 6), LB-100 (*n* = 5), anti-CAIX CAR-T (*n* = 4), and anti-CAIX CAR-T combined with LB-100 (*n* = 6). U251-Luc tumors in the respective groups were harvested two weeks after initiation of treatment and analyzed by flow cytometry. Representative FACS plots of CD4^+^ and CD8^+^ cells in tumors (A). Percentage of CD3^+^ T cells in tumors (**B**). Percentage of CD4^+^ and CD8^+^ cells in tumors (**C**). All data are shown as the mean ± SEM. * *p* < 0.05 and ** *p* < 0.01 by Student’s *t*-test, anti-CAIX CAR-T combined with LB-100 vs. anti-CAIX CAR-T group, anti-CAIX CAR-T group vs. un-treated group or LB-100 group.

**Figure 5:**
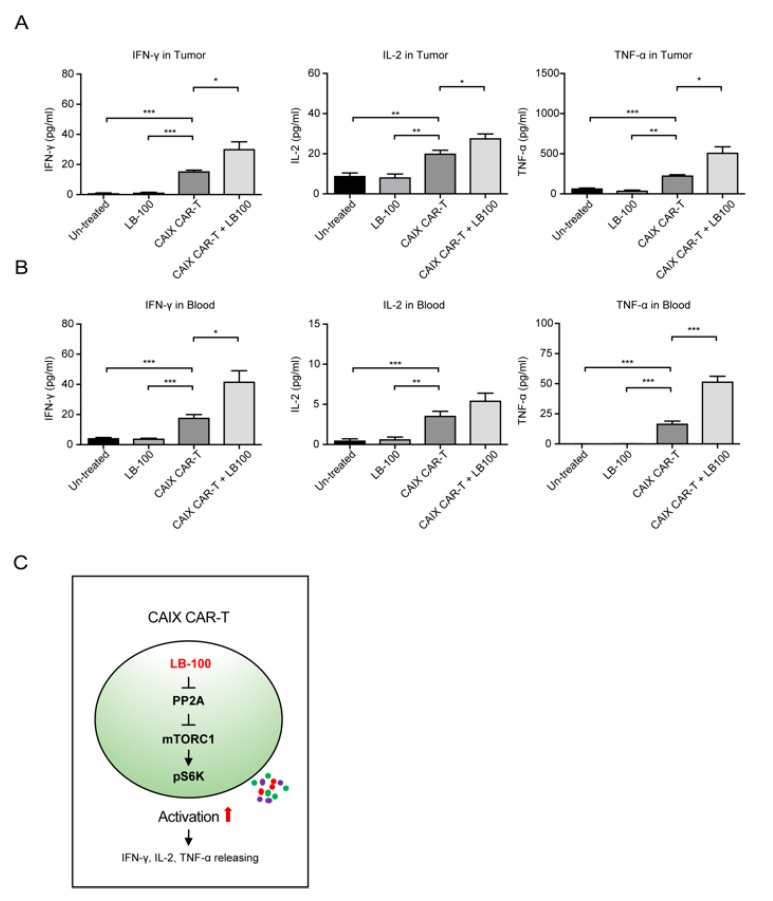
LB-100 enhances a robust CAR-T cell response. (**A**,**B**) Cytokine (IFN-γ, TNF-α, and IL-2) secretion in the supernatant of the tumor (**A**) and blood (**B**) was analyzed by ELISA. The bar graphs represent a significant increase in cytokine release in anti-CAIX CAR-T treated groups. A combination with LB-100 further enhanced cytokine release. (**C**) Mechanistic illustration of LB-100’s function in anti-CAIX CAR-T cells is shown. All data are shown as the mean ± SEM. * *p* < 0.05, ** *p* < 0.01, and *** *p* < 0.001 by Student’s *t*-test, anti-CAIX CAR-T combined with LB-100 vs. anti-CAIX CAR-T group, anti-CAIX CAR-T group vs. un-treated group or LB-100 group.

Harvested brain tumors from the LB-100 plus anti-CAIX CAR-T treatment group demonstrated a significant increase in T-lymphocytes (CD3^+^) when compared to control or single treatment groups (Figure 4A–C). Further analysis of CD8^+^ and CD4^+^ T-cell populations revealed that mice treated with both anti-CAIX CAR-T cells and LB-100 demonstrated significantly higher quantities of CD8^+^ and CD4^+^ T cells at the tumor site (Figure 4C). Of note, mice that received combination treatment demonstrated significantly higher quantities of CD8^+^ cells at the tumor site, which has been previously proven to be one of the most important predictors of response to immunotherapy [27]. In addition, we also observed that mice that received combination treatment demonstrated significantly higher levels of IFN-γ, TNF-α, and IL-2 in tumor supernatant and blood when compared to control or single treatment groups (Figure 5A,B), confirming that LB-100 increased cytokine secretion. Collectively, these data suggest that inhibition of PP2A with LB-100 significantly enhances the function of anti-CAIX CAR-T cells and together produce a synergistic anti-tumor effect against U251 glioblastoma (Figure 5C).

## 3. Discussion

We investigated the therapeutic efficacy of a combination treatment strategy consisting of anti-CAIX CAR-T cells and LB-100. We demonstrated that LB-100 significantly enhances the anti-glioma immune response of the anti-CAIX CAR-T cells in vitro and in vivo. Notably, when used in combination, anti-CAIX CAR-T cell therapy and LB-100 significantly improved tumor growth control and prolonged survival in an intracranial U251 glioblastoma model. 

The major challenges that limit the function of CAR-T cells in solid tumors include their effective localization and penetration into the tumor bed, their sub-optimal function within tumor microenvironments and their off-tumor adverse effects for targeting antigens expressed on normal tissues [28]. Anti-CAIX CAR-T cell therapy has been well studied in renal cell carcinoma (RCC) patients. However, some RCC patients developed anti-CAR T cell antibodies and cellular responses that attenuated the antitumor effects of this therapy. Unexpected on-target but off-tumor adverse effects on the bile ducts were also frequently observed due to the high CAIX expression on bile duct epithelium [29,30,31]. Recently, using a murine-based intracranial glioblastoma model, we demonstrated that in-situ injection of anti-CAIX CAR-T cells not only improved the localization and penetration of CAR-T cells but also avoided off-tumor adverse effects observed with systemic delivery of anti-CAIX CAR-T cells [14]. In the current investigation, we build on our previous findings and demonstrate that the addition of LB-100 augments the cytotoxic profile of anti-CAIX CAR-T Cells and improves their therapeutic effect in a similar intracranial glioblastoma model.

LB-100, as an inhibitor of PP2A, was demonstrated as an effective chemo- or radio-sensitizer in various preclinical studies [32]. PP2A inhibition has a dose-dependent dualistic response that can either induce apoptosis or promote cell proliferation. Low doses of PP2A inhibition have been demonstrated to promote cell proliferation, while high doses of PP2A inhibition have been shown to induce cell apoptosis [33,34]. Recently, Ho et al. (in 2018) demonstrated that a low dose of LB-100 enhanced T-cell activation and proliferation through the mTORC1 signaling pathway in a murine colon carcinoma model [19]. In our study, we used an LB-100 dose below the 1 µM concentration that did not produce an intrinsic cytotoxic effect to tumor cells nor CAR-T cells in vitro. Notably, the human equivalent dosage of LB-100 used in our in vivo mouse study is 0.5 mg/m^2^, which is below the maximally tolerated human dose of 2.33 mg/m^2^ [35]. There were no autoimmune side effects observed in a phase 1 trial with LB-100 at a similarly low dose [19,20]. We also demonstrated that a combination of CAR-T therapy with LB-100 increased PD-L1 expression on tumor cells, which suggests that the checkpoint mechanisms to suppress adaptive immune responses are still present, and that combination with checkpoint inhibitors may improve therapeutic efficacy. However, the safety and efficacy of this combination strategy remain to be elucidated. 

Our investigation demonstrates that a low dose of LB-100 can enhance the anti-CAIX CAR-T cell cytotoxicity and significantly improve survival in glioblastoma bearing mice. To our knowledge, this is the first investigation identifying a synergistic effect between CAR-T cell therapy and LB-100. 

## 4. Materials and Methods

### 4.1. Drugs

LB-100 was provided by Lixte Biotechnology, East Setauket, NY, USA, under NCI M-CRADA # 03094.

### 4.2. Cell Culture and Reagents

HEK293T cells, glioblastoma cell lines T98G, A172, and LN229 were obtained from American Type Culture Collection (ATCC; Manassas, VA, USA). The glioblastoma cell line U251 was obtained from Sigma–Aldrich (St. Louis, MO, USA). All cells were cultured in Dulbecco’s modified Eagle’s medium (DMEM; Gibco, Carlsbad, CA, USA) supplemented with 10% fetal bovine serum (FBS; Gibco) and 1% penicillin and streptomycin (Gibco). U251-Luc cells were generated by stable transduction of luciferase-containing Lentivirus (EF1a-ffLuc2-eGFP) into naïve U251 cells.

### 4.3. Generation of CAIX CAR-Expressing T Cells

The CAIX CAR-expressing vector (Lenti-EF1a-CAIX-3rd-CAR) was generated using the pLenti-EF1a-C-mGFP Tagged Cloning Vector (OriGene Technologies, Rockville, MD, USA) as previously described [14]. In brief, the mGFP sequence on the original vector was replaced by the CAR cassette, including a signal peptide, anti-CAIX single-chain variable fragment (scFv), CD8 hinge, CD28 transmembrane intracellular domain, 4-1BB, and CD3ζ. The final vector was confirmed by restriction digestion and Sanger sequencing.

Lentiviral envelope expressing plasmid pMD2.G and packaging plasmid psPAX2 were gifts from Didier Trono (Addgene plasmid # 12259 and 12260, respectively, Watertown, MA, USA). pMD2.G, psPAX2, and Lenti-EF1a-CAIX-3rd-CAR plasmids were transfected at a ratio of 2:4:5 into HEK293T cells cultured in DMEM without antibiotics. The medium was changed every day, and the supernatants were collected for the next two days. The lentiviruses were quantified using HIV-1 p24 Antigen ELISA (ZeptoMetrix, Buffalo, NY, USA) and were concentrated using Lenti-X Concentrator (Clontech Laboratories, Mountain View, CA, USA).

Peripheral blood mononuclear cells (PBMCs) were derived from healthy donors recruited by the Blood Bank, Clinical Center, NIH, and kept in liquid nitrogen until used. PBMCs were thawed in RPMI 1640 overnight and activated with Dynabeads Human T-Activator CD3/CD28 (Thermo Fisher Scientific, Waltham, MA, USA) at a ratio of 1:1 in AIM V medium (Gibco) supplemented with 5% human serum (Gibco) for 24 h. Living cells were enriched using lymphocyte separation medium and washed with phosphate-buffered saline (PBS; Gibco) twice. T cells were then transduced with lentivirus containing anti-CAIX CAR vectors or empty vectors at 1200× *g* for 2 h at 32 °C in a V-bottom 96-well plate (Corning, Corning, NY, USA). Each well contained 0.25 million cells and viruses at a multiplicity of infection (MOI) of 40, with 8 µg/mL polybrene (Sigma-Aldrich, St. Louis, MO, USA) and 300 international units (IU) human interleukin 2 (hIL-2; Peprotech, Rocky Hill, NJ, USA). Transduced cells were resuspended after 3 h and were transferred to a 6-well plate for expansion in the presence of 100 IU hIL-2 for two to three days.

### 4.4. Cell Viability Assay

Cell viability of glioblastoma cells and T cells treated with LB-100 alone was assessed with a CCK8 assay kit (Dojindo, Tabaru, Japan). Approximately 5 × 10^3^ glioblastoma cells or 2 × 10^4^ T cells were seeded into each well of 96-well plates. After culturing overnight, the cells were treated with titration concentrations of LB-100. After a 48 h treatment, CCK-8 solution was added to give a final concentration of 1 mg/mL. Absorbance values were determined at 450 nm with Synergy H1 Hybrid Multi-Mode Reader (BioTek, Winooski, VT, USA) after 2 h incubation. All the CCK8 assays were performed in four replicates.

The LDH assay was performed to detect the in vitro cytotoxic effect of anti-CAIX CAR-T cells on glioblastoma cells with an LDH Cytotoxicity Assay Kit (Cayman Chemical, Ann Arbor, MI, USA). 5 × 10^3^ glioblastoma cells were seeded in 96-well plates and incubated at 37 °C overnight. Anti-CAIX CAR-T cells were added to glioblastoma cells at the ratio of 4:1. After 48-h of incubation, the plates were centrifuged at 400× *g* for 5 min. Fifty microliters of supernatant was moved to a new 96-well plate, and 50 µL of LDH Reaction Solution was added. After incubation at 37 °C for 30 min, the absorbance value at 490 nm was read for statistical analysis. All the LDH assays were performed in triplicates.

### 4.5. Immunoblotting 

Immunoblotting was performed as previously described [36]. Briefly, 1 × 10^6^ glioblastoma cells were seeded in 6-well plates and incubated at 37 °C overnight. T cells were added to glioblastoma cells at the ratio of 4:1. After 48 h of incubation, T cells were washed out with PBS, and proteins were collected from left tumor cells. A total of 40 µg of proteins were subjected to electrophoresis and were transferred to a nitrocellulose membrane. After blocking with 5% non-fatty milk, the membrane was incubated with primary antibodies (1:1000 dilution) at 4 °C overnight, followed by incubation of secondary antibodies (1:3000 dilution; from Cell Signal Technology, Danvers, MA, USA). The anti-pdl1 antibody was purchased from Novus Biologicals (Littleton, CO, USA) [37].

### 4.6. Enzyme-Linked Immune Sorbent Assay (ELISA)

Cells were treated as indicated for 48 h, and supernatants were collected. Cells and cell debris were removed from samples by centrifugation at 5000× *g* for 5 min, and the samples were kept at −80 °C until used. Blood samples from mice were collected into tubes with EDTA from the orbital sinus, and then the blood cells were removed by centrifugation at 10,000× *g* for 10 min, and the plasmas were stored at −80 °C until used. Concentrations of IFN-γ, TNF-α, and IL-2 were determined using Human IFN-γ ELISA Kit II, Human TNF-α ELISA Kit II, and Human IL-2 ELISA Kit II (BD Biosciences, San Jose, CA, USA), respectively, according to the manufactures’ instructions.

### 4.7. Flow Cytometry

Cells were treated as indicated and then were harvested as previously described [19]. For detecting CAR scFv, biotinylated Protein L (GenScript, M00097, Piscataway, NJ, USA) was used, followed by phycoerythrin (PE)-conjugated streptavidin (SA) (Biolegend, #405203, San Diego, CA, USA) as described previously [38]. Cells were incubated with primary antibodies for 1 h at 4 °C in the dark before proceeding to secondary staining. For extracellular and secondary staining, cells were washed twice prior to 30 min incubation at 4 °C. For intracellular staining, cells were fixed, permeabilized, and processed according to the manufacturer’s protocol (BD Biosciences, San Jose, CA, USA). Cells were then incubated with fluorophore-conjugated antibodies for 30 min at 4 °C in the dark and washed twice prior to resuspension in FACS buffer. The following antibodies against human molecules were used: Brilliant violet 421 conjugated-anti-PD-1 (Biolegend, #329919), APC conjugated-anti-PD-L1 (Biolegend, #329707), and anti-Phospho-S6 ribosomal protein (Ser235/236) (D57.2.2E, Cell Signal Technology). Cells were subjected to flow cytometry using a BD FACS Canto II Flow Cytometer (BD Biosciences) as previously described [39]. Data were analyzed using FlowJo software (FlowJo, Ashland, OR, USA).

### 4.8. Xenograft Mouse Model

Mice experiments were approved by the NINDS and National Cancer Institute (NCI, Bethesda, MD, USA) Animal Use and Care Committees (Protocol No., NOB-020). NOD-*Prkdc^scid^Il2rg^tmiWjl^* (NSG) mice (6–8 weeks old from NCI-Frederick animal facility, Frederick, MD, USA) were intracranially inoculated with 100,000 U251-Luc cells suspended in 2 µL Hank’s balanced salt solution (HBSS; Crystalgen, Commack, NY, USA) using a stereotactic device (coordinates, 2 mm anterior and 2 mm lateral from bregma, and 2 mm depth from the dura). After one week, luciferin signals were detected to confirm the survival of tumor cells in mice. The mice were assigned to the indicated groups according to the signal intensity to keep the baseline balanced. A total of 2 million anti-CAIX CAR-T cells in 2 to 2.5 µL HBSS were injected into the same coordinate as tumor cell injection every week for 3 times. Untreated mice received an injection of the same volume of HBSS. LB-100 was intraperitoneally injected daily at a dose of 0.167 mg/kg body weight for 28 days. The viability of tumors was monitored every four days. Survival endpoint for all animal studies were defined as when any of the following criteria was reached: (1) a loss of more than 15% of body weight, (2) protruded skull, (3) head tile, (4) hunched posture, (5) ataxia, (6) rough hair coat, or (7) impaired mobility.

### 4.9. Isolation of Tumor-Infiltrating Lymphocytes

Mice were intracranially inoculated with 100,000 U251-Luc cells suspended in 2 µL HBSS and treated as above one week after implantation. Mice were sacrificed, and tumors excised after two-weeks of treatment. Tumors were subjected to mechanical disruption using a GentleMACS Dissociator (Miltenyi Biotec, Gaithersburg, MD, USA) in the presence of enzymatic digestion using a tumor dissociation kit (Miltenyi Biotec). The supernatant was harvested after a brief spin. Cells and cell debris were further removed from the supernatant by centrifugation at 10,000× *g* for 10 min, and the samples were kept at −80 °C until ELISA analysis. Suspensions containing T cells were stained with anti-human CD3 (#317332), CD4 (#300514), CD8 (#301032) antibodies (Biolegend, San Diego, CA, USA) in FACS buffer and then analyzed by a BD FACS Canto II Flow Cytometer (BD Biosciences, San Jose, CA, USA). Data analysis was performed using FlowJo software (FlowJo, Ashland, OR, USA).

### 4.10. PP2A Phosphatase Assay 

PP2A phosphatase assay kit (Millipore, Burlington, MA, USA) was used according to the manufacturer’s instructions. Briefly, using the same amount of starting protein lysate for each condition, PP2A was immunoprecipitated using Anti-PP2A, C subunit (clone 1D6, Millipore), and Protein A agarose slurry. The slurry was then washed with TBS before a standard amount of threonine phosphopeptide, a substrate of PP2A, was added to the mixture. Phosphate was released as a product of the reaction. The absolute amount of phosphate released was quantified with malachite green solution, which was used as a measure of PP2A activity. Experiments were performed in triplicate, and the data are presented as a percentage mean of relative PP2A activity compared with control ± SEM.

### 4.11. Statistical Analysis

Data were presented as the mean and standard deviation (SD) or standard error of the mean (SEM), as indicated. Survival curves were generated using the Kaplan–Meier estimate and were compared using a log–rank test. Survival curves were compared using a log–rank test. Other variables were analyzed using two-way ANOVA or unpaired Student’s *t*-test, as appropriate. Statistical analysis was performed using Prism 6 (GraphPad Software, San Diego, CA, USA). A *p* < 0.05 was considered as statistically significant.

## 5. Conclusions

We report on a unique combination treatment strategy to augment the therapeutic efficacy of anti-CAIX CAR-T cells against glioblastoma. The therapeutic strategy validated the role of LB-100 in augmenting the cytotoxic activity of anti-CAIX CAR-T cells within the glioblastoma microenvironment and resulted in significantly improved tumor control and survival. Notably, our findings highlight the therapeutic value in adopting combinatorial treatment strategies to address unique tumor escape mechanisms associated with solid tumors. Although our investigation into combinational CAR-T therapy was advanced in an intracranial murine glioblastoma model, we see great translational potential for adapting the aforementioned treatment strategies to the clinical setting and to the treatment of other solid tumors.

## Supplementary Materials

The following are available online at https://www.mdpi.com/2072-6694/12/1/139/s1, Figure S1: Effect of LB-100 on anti-CAIX CAR-T cells, Figure S2: Effect of LB-100 on glioblastoma cells, Figure S3: LB-100 enhances cytotoxicity of anti-CAIX CAR-T cells against glioblastoma in vitro, Figure S4: Original uncut gels of the cropped gels in Figure 1E, Figure S5: Anti-CAIX CAR-T cells increase stable PD-L1 expression on tumor cells and CAR-T cells.
