# Supplementary material for: Inhibition of PP2A with LB-100 Enhances Efficacy of CAR-T Cell Therapy Against Glioblastoma
Source: Cancers (Basel). 2020 Jan 6;12(1):139. doi: 10.3390/cancers12010139 (PMC7017120; doi:10.3390/cancers12010139)
Supplement: Supplementary file 1 [file cancers-12-00139-s001.pdf]

Supplementary Materials

Inhibition of PP2A with LB-100 Enhances Efficacy of CAR-T Cell Therapy Against Glioblastoma

Jing Cui, Herui Wang, Rogelio Medina, Qi Zhang, Chen Xu, Iris H. Indig, Jingcheng Zhou, Qi Song, Pauline Dmitriev, Mitchell Y. Sun, Liemei Guo, Yang Wang, Jared S. Rosenblum, John S. Kovach, Mark R. Gilbert and Zhengping Zhuang

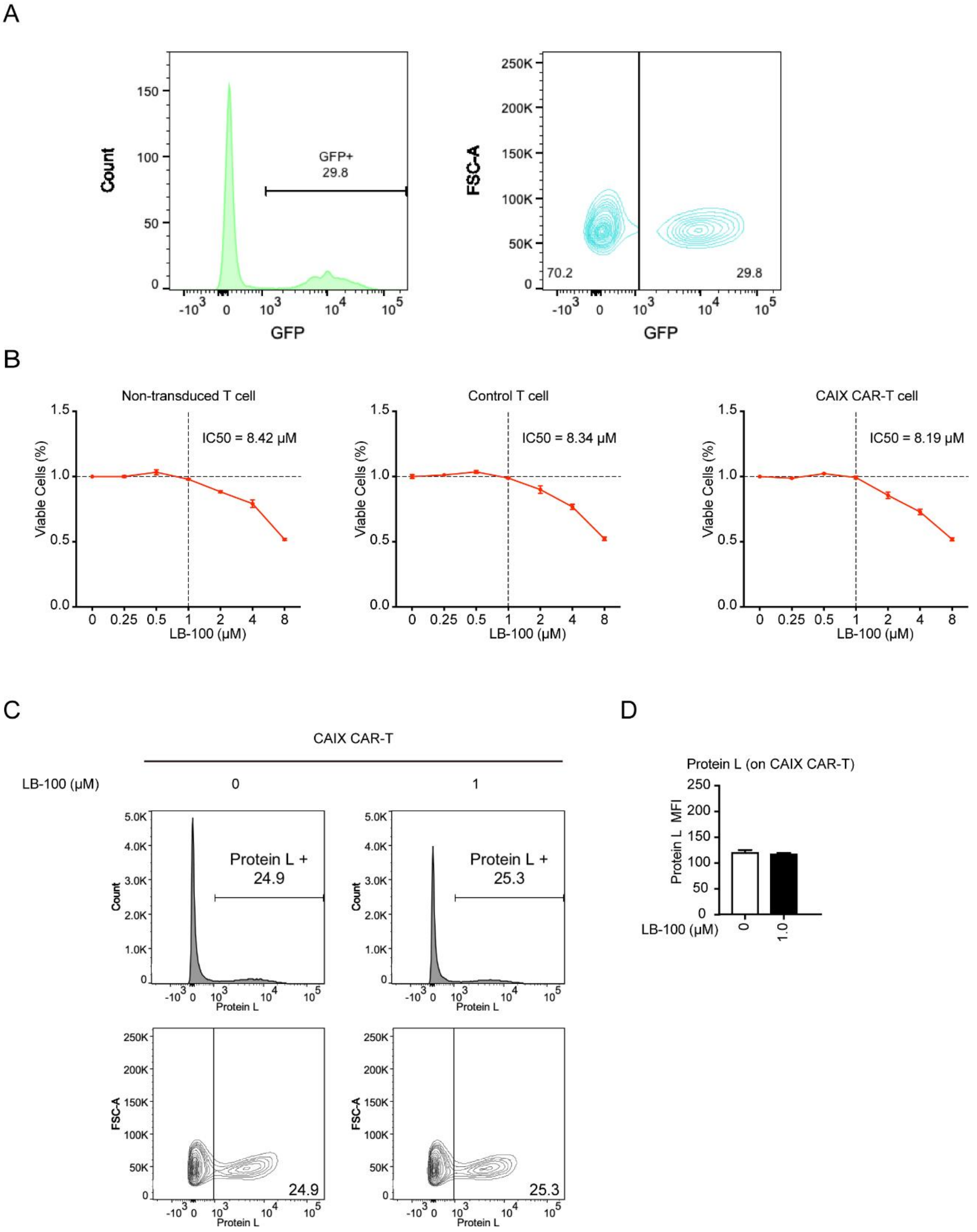

Figure S1. Effect of LB-100 on anti-CAIX CAR-T cells. (**A**) Transduction efficiency was detected by green fluorescent protein (GFP) expression in mock T cells (using GFP instead of CAIX CAR scFv) on day 4 post-transduction using flow cytometry. The transduction efficiency was around 30%. (**B)**

CCK-8 results showed LB-100 dose-response curve of non-transduced T, control T (empty vector transduced), and anti-CAIX CAR-T cells for 48 hours. IC50 of each cell line was calculated and listed as indicated. (**C**) Flow cytometry analyzing protein L (CAR scFv) expression on control T cells and anti-CAIX CAR-T cells in presence of 1 μM LB-100. (**D**) LB-100 has no effect on CAR expression.

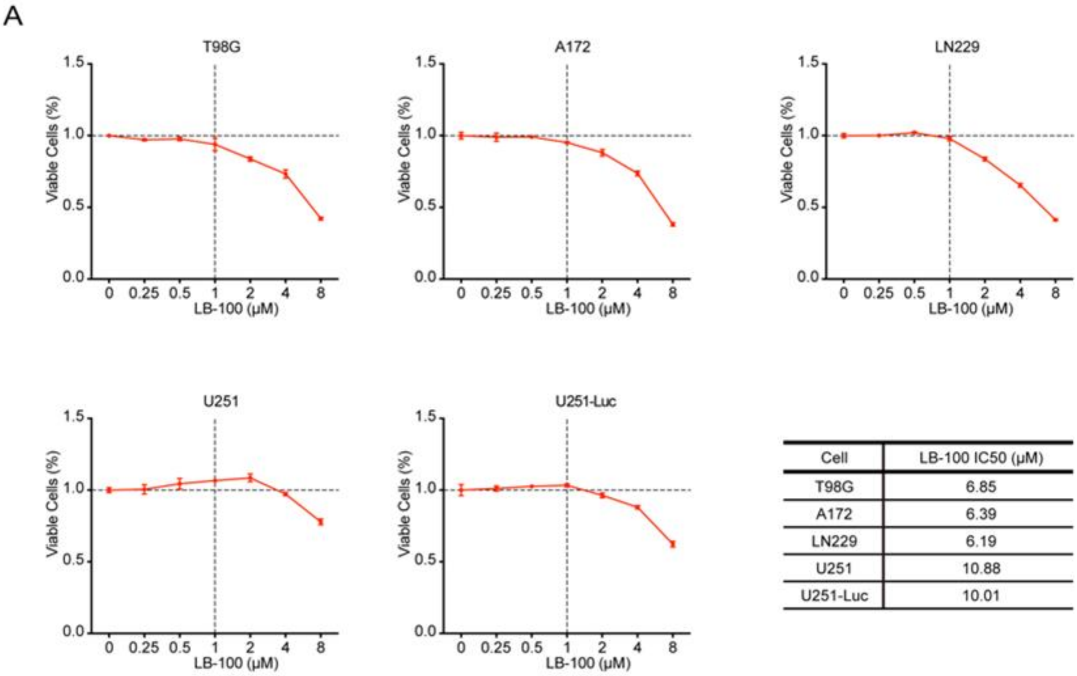

Cell	LB-100 IC50 (μM)
T98G	6.85
A172	6.39
LN229	6.19
U251	10.88
U251-Luc	10.01

Figure S2. Effect of LB-100 on glioblastoma cells. (**A**) CCK-8 assay results showed the LB-100 dose-response curve of glioblastoma cells (T98G, A172, LN229, U251 and U251-Luc) for 48 hours. IC50 of each cell line was calculated and listed as indicated.

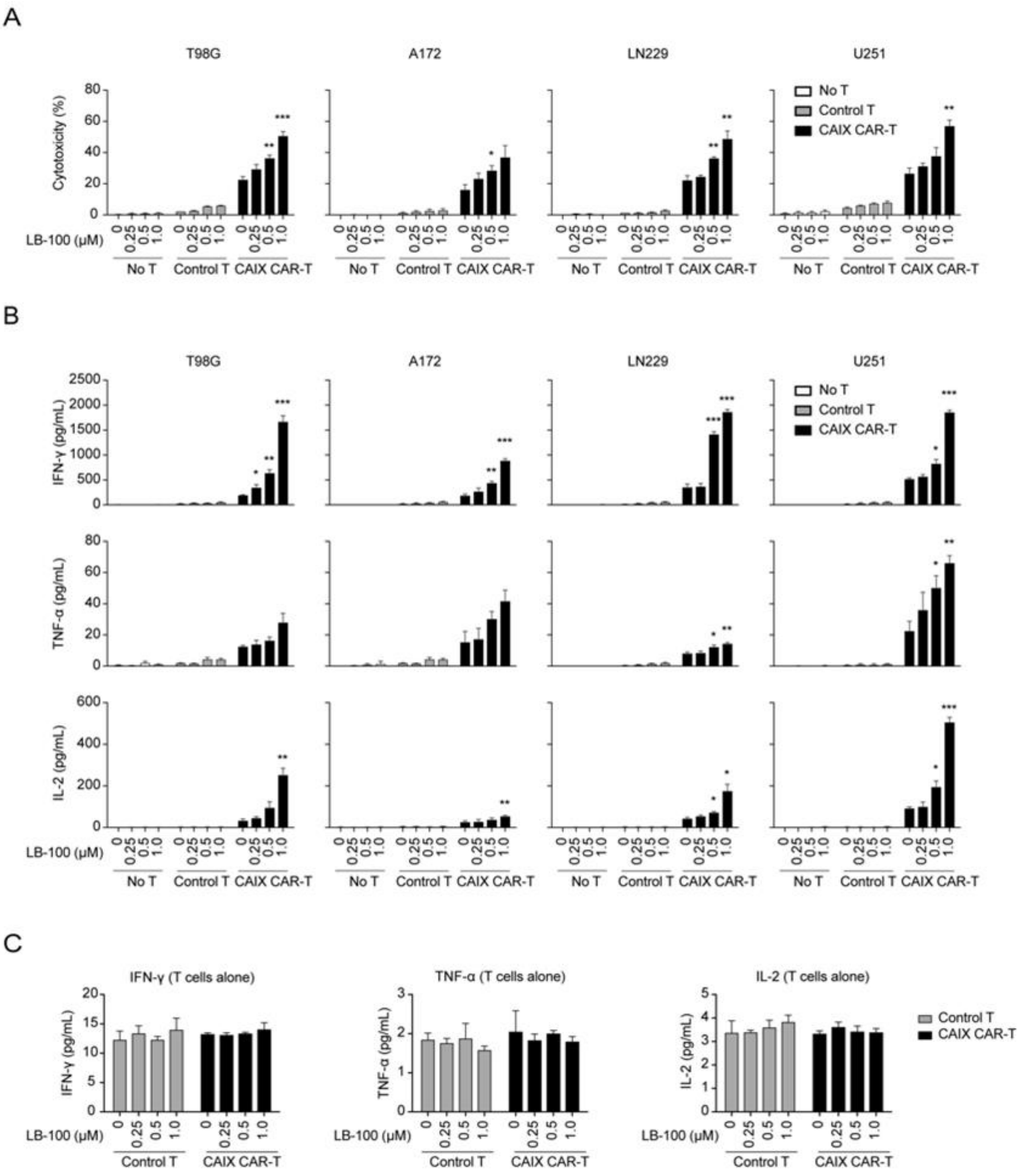

Figure S3. LB-100 enhances cytotoxicity of anti-CAIX CAR-T cells against glioblastoma in vitro. (**A**) Control T cells (empty vector transduced T cells) or anti-CAIX CAR-T cells were cocultured with glioblastoma cells (T98G, A172, LN229, and U251) with titration concentration of LB-100 as indicated (1 μM) at an E/T ratio at 4 for 48 hours. Cytotoxicity was determined by LDH releasing assay. $n = 3$ for each group. (**B**) Cytokine (IFN-γ, TNF-α, and IL-2) secretion in the supernatant obtained from the cocultured system was analyzed by ELISA. The bar graphs represent a significant increase in cytokine release in anti-CAIX CAR-T treated groups. A combination of LB-100 further enhanced cytokine release. (**C**) Cytokine (IFN-γ, TNF-α, and IL-2) secretion in the supernatant obtained from control T cells or anti-CAIX CAR-T cells cultured alone in the presence of LB-100 was analyzed by ELISA. Levels of cytokines were comparable in anti-CAIX CAR-T treated groups and in the combination groups. All data are shown as the mean ± SEM. * $p < 0.05$, ** $p < 0.01$, and *** $p < 0.001$ by Student's *t*-test, anti-CAIX CAR-T combined with LB-100 groups vs. anti-CAIX CAR-T group.

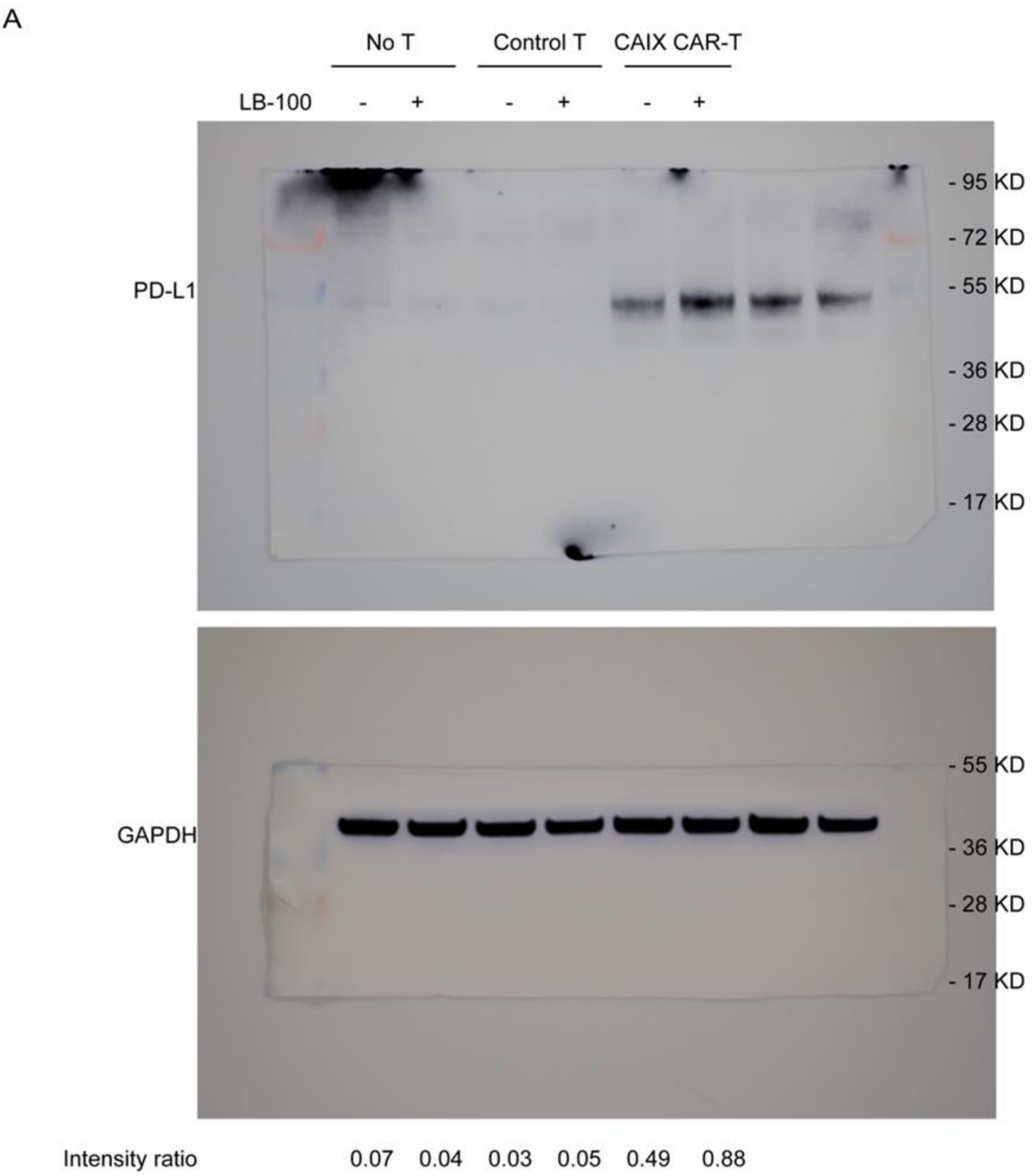

Figure S4. Original uncut gels of the cropped gels in Figure 1E.

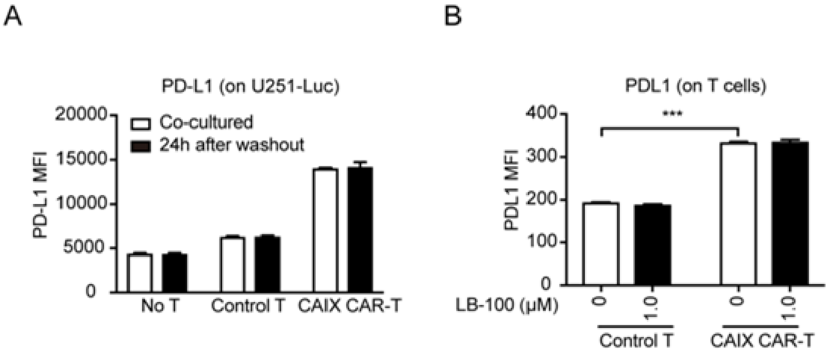

Figure S5. Anti-CAIX CAR-T cells increase stable ligand programmed death-1 (PD-L1) expression on tumor cells and CAR-T cells. (**A**) U251-Luc cells were treated with control-T or anti-CAIX CAR-T cells at an E/T ratio of 4 for 48 hours, followed by washout for 24 hours; un-treated U251-Luc cells

served as control. PD-L1 expression on U251-Luc cells in each group was determined by flow cytometry as indicated. There is no significant difference of mean fluorescence intensity (MFI) of PD-L1 positive cells in CAR-T cell treated U251-Luc cells before and after CAR-T cell washout (n = 3). (**B**) Control T cells (empty vector transduced T cells) or anti-CAIX CAR-T cells were co-cultured with U251-Luc cells with 1 μM LB-100 at an E/T ratio at 4 for 48 hours (n = 3). Flow cytometry analyzing PD-L1 expression on control-T or anti-CAIX CAR-T cells from co-cultured system. There is a significant increase in MFI of PD-L1 positive cells in anti-CAIX CAR-T cells compared with control T cells, but no further increase in the combination groups. All data are shown as the mean ± SEM. *** $p < 0.001$ by Student's t-test; anti-CAIX CAR-T groups vs. control T groups.

© 2020 by the authors. Licensee MDPI, Basel, Switzerland. This article is an open access article distributed under the terms and conditions of the Creative Commons Attribution (CC BY) license (http://creativecommons.org/licenses/by/4.0/).
